# Downregulation of miR-141-3p promotes bone metastasis via activating NF-κB signaling in prostate cancer

**DOI:** 10.1186/s13046-017-0645-7

**Published:** 2017-12-04

**Authors:** Shuai Huang, Qingde Wa, Jincheng Pan, Xinsheng Peng, Dong Ren, Yan Huang, Xiao Chen, Yubo Tang

**Affiliations:** 1 Department of Orthopaedic Surgery, the Second Affiliated Hospital of Guangzhou Medical University, 510260 Guangzhou, People’s Republic of China; 2 Department of Orthopaedic Surgery, the First Affiliated Hospital of Sun Yat-sen University, 510080 Guangzhou, People’s Republic of China; 3 Department of Orthopaedic Surgery, the Affiliated Hospital of Zunyi Medical college, 563003 Zunyi, People’s Republic of China; 4 Department of Urology Surgery, the First Affiliated Hospital of Sun Yat-sen University, 510080 Guangzhou, People’s Republic of China; 5 Department of Pharmacy, The First Affiliated Hospital of Sun Yat-Sen University, 58 Zhongshan 2nd Road, Guangzhou, Guangdong 510080 People’s Republic of China

**Keywords:** miR-141-3p, EMT, Bone metastasis, NF-κB signaling and prostate cancer

## Abstract

**Background:**

Clinically, prostate cancer (PCa) exhibits a high avidity to metastasize to bone. miR-141-3p is an extensively studied miRNA in cancers and downregulation of miR-141-3p has been widely reported to be involved in the progression and metastasis of several human cancer types. However, the clinical significance and biological roles of miR-141-3p in bone metastasis of PCa are still unclear.

**Methods:**

miR-141-3p expression was examined in 89 non-bone metastatic and 52 bone metastatic PCa tissues by real-time PCR. Statistical analysis was performed to investigate the clinical correlation between miR-141-3p expression levels and clinicopathological characteristics in PCa patients. The biological roles of miR-141-3p in bone metastasis of PCa were evaluated both in vitro and a mouse intracardial model in vivo. Bioinformatics analysis, Western blot, luciferase reporter and miRNA immunoprecipitation assays were performed to explore and examine the relationship between miR-141-3p and its potential targets. Clinical correlation of miR-141-3p with its targets was examined in clinical PCa tissues.

**Results:**

miR-141-3p expression is reduced in bone metastatic PCa tissues compared with non-bone metastatic PCa tissues. Low expression of miR-141-3p positively correlates with serum PSA levels, Gleason grade and bone metastasis status in PCa patients. Furthermore, upregulating miR-141-3p suppresses the EMT, invasion and migration of PCa cells in vitro. Conversely, silencing miR-141-3p yields an opposite effect. Importantly, upregulating miR-141-3p dramatically reduces bone metastasis of PC-3 cells in vivo. Our results further show that miR-141-3p inhibits the activation of NF-κB signaling via directly targeting tumor necrosis factor receptor-associated factor 5(TRAF5) and 6 (TRAF6), which further suppresses invasion, migration and bone metastasis of PCa cells. The clinical negative correlation of miR-141-3p expression with TRAF5, TRAF6 and NF-κB signaling activity is demonstrated in PCa tissues.

**Conclusion:**

Our findings unravel a novel mechanism underlying the bone metastasis of PCa, suggesting that miR-141-3p mimics might represent a potential therapeutic avenue for the treatment of PCa bone metastasis.

**Electronic supplementary material:**

The online version of this article (10.1186/s13046-017-0645-7) contains supplementary material, which is available to authorized users.

## Background

Prostate cancer (PCa) is the most frequently diagnosed malignancy and the second leading cause of cancer-related deaths in men [1]. Despite great advances in systemic and individualized treatments of PCa in the last decades, the incidence of PCa remains markedly increasing in China [2, 3]. The skeleton is the most common site for PCa metastases, which severely affects the quality of life and survival time of PCa patients [4]. Therefore, better understanding of the underlying mechanisms responsible for bone metastasis of PCa facilitates the identification of novel therapeutic targets for bone metastasis of PCa.

Since identified in several decades ago [5], the central roles of the NF-κB pathway in physiologic and pathologic processes, including inflammation and tumorigenesis, have been well documented [6, 7]. Constitutive activation of NF-κB signaling has been reported in numerous human cancers, which promotes the initiation, progression and metastasis of malignancies [8–12]. Notably, ubiquitination- and phosphorylation-mediated signaling transduction has been identified as an important regulatory mechanism for the activation of NF-kB signaling [13, 14]. After binding to respective ligands, the receptors recruit multiple receptor-associated factors, such as tumor necrosis factor receptor-associated factor (TRAFs), function as a ubiquitin ligase via inducing the K-63 polyubiquitination of receptor-interacting protein 1 (RIP1), resulting in activation of transforming growth factor β–activated kinase-1 (TAK1)/TAB2/3 complex. Activated TAK1/TAB2/3 complex phosphorylates and activates inhibitor of NF-kB kinase (IKK)-α/β/γ complex, leading to nuclear translocation and activation of NF-kB [14, 15]. Furthermore, several lines of evidence have reported that NF-κB signaling plays a critical role in the bone metastasis of cancers [9, 16, 17]. Importantly, NF-κB activation has also been reported to be associated with the metastatic phenotype of PCa progression [18, 19]. A study by Chen et al. reported that NF-κB signaling activity promoted the development of PCa bone metastasis [19]. However, the underlying mechanism of constitutive activation of NF-κB signaling in the bone metastasis of PCa needs to be further elucidated.

MicroRNAs (miRNAs) are a series of small non-coding RNAs with 18–24 nucleotides that post-transcriptionally regulate target genes via binding to specific sequences in the 3′ untranslated region (3’UTR) of downstream target genes, leading to mRNA degradation and/or translational inhibition [20]. Abundant evidence has revealed that the aberrant expression of miRNAs was associated with progressive and metastatic phenotypes of cancers [21–24]. Our previous studies in combination with other literatures have shown that several dysregulation of miRNAs were crucial mediators in the bone metastasis of PCa [25–28]. As one of the originally discovered miRNAs, dysregulation of miR141-3p is implicated in the progression and metastasis of various cancers [29, 30].Importantly, a study by Liu and colleagues has demonstrated that bone metastatic PCa cells PC-3 expressed little endogenous miR-141-3p [29], suggesting that low expression of miR-141-3p may play an important role in the bone metastasis of PCa. However, the clinical significance and biological roles of miR-141-3p in the bone metastasis of PCa remain unclear.

In this study, we report that miR-141-3p is downregulated in bone metastatic PCa tissues compared with non-bone metastatic PCa tissues. miR-141-3p expression inversely correlates with the clinicopathological characteristics and bone metastasis status in PCa patients. Furthermore, upregulating miR-141-3p represses, while silencing miR-141-3p enhances EMT, invasion and migration of PCa cells in vitro. Importantly, upregulating miR-141-3p significantly inhibits bone metastasis of PC-3 cells in vivo. Our results further demonstrate that ectopic expression of miR-141-3p suppresses activity of NF-κB signaling via targeting TRAF5 and TRAF6, which further inhibits invasion, migration and bone metastasis in PCa. The analysis of clinical correlation shows that miR-141-3p inversely correlates with TRAF5 and TRAF6 expression, as well as with NF-κB signaling activity and downstream target genes of NF-κB signaling in human PCa tissues. Taken together, these findings clarify a novel mechanism responsible for constitutive activation of NF-κB signaling in bone metastasis of PCa, determining that miR-141-3p play a tumor-suppressive role in bone metastasis of PCa.

## Methods

### Cell culture

The human PCa cell lines 22RV1, PC-3, VCaP, DU145, LNCaP and normal prostate epithelial cells RWPE-1 were obtained from Shanghai Chinese Academy of Sciences cell bank (China). RWPE-1 cells were grown in defined keratinocyte-SFM (1×) (Invitrogen). PC-3, LNCaP and 22Rv1 cells were cultured in RPMI-1640 medium (Life Technologies, Carlsbad, CA, US) supplemented with penicillin G (100 U/ml), streptomycin (100 mg/ml) and 10% fetal bovine serum (FBS, Life Technologies). DU145 and VCaP cells were grown in Dulbecco’s modified Eagle’s medium (Invitrogen) supplemented with 10% FBS. The C4-2B cell line was purchased from the MD Anderson Cancer Center and maintained in T-medium (Invitrogen) supplemented with 10% FBS. All cell lines were grown under a humidified atmosphere of 5% CO2 at 37 °C.

### Plasmid, small interfering RNA and transfection

The human miR-141-3p gene was PCR-amplified from genomic DNA and cloned into a pMSCV-puro retroviral vector (Clontech, Japan). The pNFκB-luc and control plasmids (Clontech, Japan) were used to examine the activity of transcription factor quantitatively. The 3′-untranslated region (3’UTR) regions of the human TRAF5 and TRAF6 were PCR-amplified from genomic DNA and cloned into pmirGLO vectors (Promega, USA), and the list of primers used in cloning reactions is presented in Additional file 1: Table S1. Anti-miR-141-3p, small interfering RNA (siRNA) for the TRAF5 and TRAF6 knockdown and corresponding control siRNAs were synthesized and purified by RiboBio. Transfection of miRNA, siRNAs, and plasmids was performed using Lipofectamine 3000 (Life Technologies, USA) according to the manufacturer’s instructions.

### RNA extraction, reverse transcription, and real-time PCR

Total miRNA from tissues or cells was extracted using the mirVana miRNA Isolation Kit (Ambion). Messenger RNA (mRNA) and miRNA were reverse transcribed from total mRNA using the RevertAid First Strand cDNA Synthesis Kit (Thermo Fisher, USA) according to the manufacturer’s protocol. Complementary DNA (cDNA) was amplified and quantified on the CFX96 system (BIO-RAD, USA) using iQ SYBR Green (BIO-RAD, USA). The primers are provided in Additional file 2: Table S2. Real-time PCR was performed according to a standard method, as described previously [31]. Primers for U6 and miR-141-3p were synthesized and purified by RiboBio (Guangzhou, China). U6 or glyceraldehyde-3-phosphate dehydrogenase (GAPDH) was used as the endogenous controls. Relative fold expressions were calculated with the comparative threshold cycle (2^-ΔΔCt^) method.

### Western blotting

Nuclear/cytoplasmic fractionation was separated using the Cell Fractionation Kit (Cell Signaling Technology, USA) according to the manufacturer’s instructions, and the whole cell lysates were extracted with RIPA Buffer (Cell Signaling Technology). Western blotting was performed according to a standard method, as described previously [32]. Antibodies against E-cadherin (Cat# 3195), Vimentin (Cat# 5741), Fibronectin (Cat# 4706), TRAF1 (Cat# 4710), TRAF5 (Cat# 41658) and TRAF6 (Cat# 8028) were purchased from Cell Signaling Technology, p65 (cat# 10745–1-AP) from Proteintech, and p84 (Cat#:PA5–27816) from Invitrogen. The membranes were stripped and reprobed with an anti–α-tubulin antibody (Sigma-Aldrich, USA) as the loading control.

### Luciferase assay

Cells (4 × 10^4^) were seeded in triplicate in 24-well plates and cultured for 24 h and performed as previously described [33]. Cells were transfected with 100 ng of the pNFκB reporter luciferase plasmid, or pmirGLO-TRAF5–3′UTR, or –TRAF6–3′UTR luciferase plasmid, plus 5 ng pRL-TK the Renilla plasmid (Promega) using Lipofectamine 3000 (Invitrogen) according to the manufacturer’s recommendations. Luciferase and Renilla signals were measured 36 h after transfection using a Dual Luciferase Reporter Assay Kit (Promega) according to the manufacturer’s protocol.

### miRNA immunoprecipitation

Cells were co-transfected with HA-Ago2, followed by HA-Ago2 immunoprecipitation using anti-HA-antibody, as previously described [34]. Real-time PCR analysis of the IP material was performed to test the association of the mRNA of SOCS1 and TNIP1 with the RISC complex.

### Invasion and migration assays

The invasion and migration assays were performed using Transwell chamber consisting of 8-mm membrane filter inserts (Corning) with or without coated Matrigel (BD Biosciences) respectively as described previously [35]. Briefly, the cells were trypsinized and suspended in serum-free medium. Then, 1.5 × 10^5^ cells were added to the upper chamber, and lower chamber was filled with the culture medium supplemented with 10% FBS. After incubation for 24–48 h, cells passed through the coated membrane to the lower surface, where cells were fixed with 4% paraformaldehyde and stained with haematoxylin. The cell count was performed under a microscope (×100).

### Animal study

All mouse experiments were approved by The Institutional Animal Care and Use Committee of Sun Yat-sen University and the approval-No. was L102012016110D. For the bone metastasis study, BALB/c-nu mice ((5–6 weeks old, 18–20 g)) were anaesthetized and inoculated into the left cardiac ventricle with 1 × 10^5^ PC-3 cells in 100 μl of PBS. Bone metastases were monitored by bioluminescent imaging (BLI) as previously described [36]. Osteolytic lesions were identified on radiographs as radiolucent lesions in the bone. The area of the osteolytic lesions was measured using the Metamorph image analysis system and software (Universal Imaging Corporation), and the total extent of bone destruction per animal was expressed in square millimeters. Each bone metastasis was scored based on the following criteria: 0, no metastasis; 1, bone lesion covering <1/4 of the bone width; 2, bone lesion involving 1/4~1/2 of the bone width; 3, bone lesion across 1/2~3/4 of the bone width; and 4, bone lesion >3/4 of the bone width. The bone metastasis score for each mouse was the sum of the scores of all bone lesions from four limbs. For survival studies, mice were monitored daily for signs of discomfort, and were either euthanized all at one time or individually when presenting signs of distress, such as a 10% loss of body weight, paralysis, or head tilting.

### Patients and tumor tissues

A total of 141 archived PCa tissues, including 89 non-bone metastatic PCa tissues and 52 bone metastatic PCa tissues were obtained during surgery or needle biopsy at The First People’s Hospital of Guangzhou City (Guangzhou, China) between January 2008 and October 2016. Patients were diagnosed based on clinical and pathological evidence, and the specimens were immediately snap-frozen and stored in liquid nitrogen tanks. For the use of these clinical materials for research purposes, prior patient’ consents and approval from the Institutional Research Ethics Committee were obtained. The clinicopathological features of the patients are summarized in Additional file 3: Table S3. The median of miR-141-3p expression in PCa tissues was used to stratify high and low expression of miR-141-3p.

### Statistical analysis

All values are presented as the mean ± standard deviation (SD). Significant differences were determined using the GraphPad 5.0 software (USA). Student’s t-test was used to determine statistical differences between two groups. The chi-square test was used to analyze the relationship between miR-141-3p expression and clinicopathological characteristics. *P* < 0.05 was considered significant. All experiments were repeated three times.

## Results

### miR-141-3p is downregulated in bone-metastatic PCa tissues

**Fig. 1:**
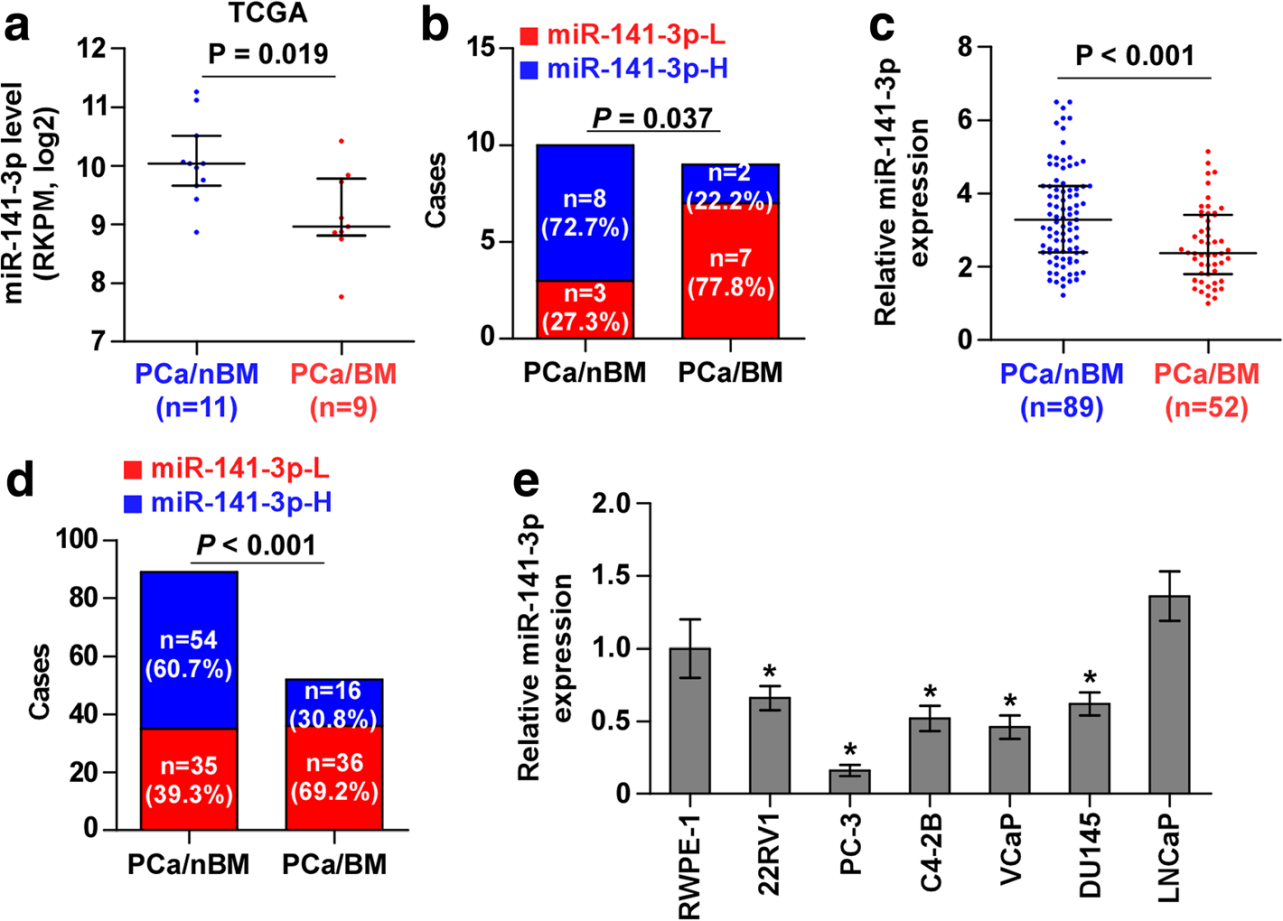
miR-141-3p is downregulated in bone metastatic PCa tissues and cells. **a** miR-141-3p expression levels was decreased in bone metastatic PCa tissues (PCa/BM) compared with that in non-bone metastatic PCa tissues (PCa/nBM) as assessed by analyzing the TCGA PCa miRNA sequencing dataset (PCa/nBM, *n* = 11; PCa/BM, *n* = 9). **b** Percentages and number of samples showed high or low miR-141-3p expression in bone metastatic and non-bone metastatic PCa tissues in PCa dataset from TCGA. **c** Real-time PCR analysis of miR-141-3p expression in 89 non-bone metastatic and 52 bone metastatic PCa samples. Transcript levels were normalized to *U6* expression. **d** Percentages and number of samples showed high or low miR-141-3p expression in bone metastatic and non-bone metastatic PCa tissues in our PCa tissues. **e** Real-time PCR analysis of miR-141-3p expression levels in normal prostate epithelial cell (RWPE-1), primary PCa cell 22RV1, bone metastatic PCa cell lines (PC-3, C4-2B and VCaP) and brain metastatic cell line DU145 and lymph node metastatic cell line LNCaP. Transcript levels were normalized to *U6* expression. *P < 0.05

Through analyzing the miRNA sequencing dataset of PCa from The Cancer Genome Atlas (TCGA), we found that miR-141-3p expression was downregulated in bone metastatic PCa tissues compared with that in non-bone metastatic PCa tissues (Fig. 1a), and the percentage of low expression of miR-141-3p was higher in bone metastatic PCa tissues than that in non-bone metastatic PCa tissues (Fig. 1b).We further examined the expression levels of miR-141-3p in our PCa tissues and found that the miR-141-3p expression level in bone metastatic PCa tissues was dramatically decreased compared with that in non-bone metastatic PCa tissues (Fig. 1c), and the percentage of low expression of miR-141-3p was higher in bone metastatic PCa tissues than that in non-bone metastatic PCa tissues (Fig. 1d). Consistently, miR-141-3p expression was differentially downexpressed in PCa cells compared with normal prostate epithelial cells RWPE-1, except for lymph node metastatic cell line LNCaP, and the lowest expression level of miR-141-3p was observed in bone metastatic PCa cell lines PC-3 (Fig. 1e). Statistical analysis of PCa tissue samples demonstrated that low expression of miR-141-3p positively correlated with serum PSA levels, Gleason grade and bone metastasis status in PCa patients (Additional file 3: Table S3 and Additional file 4: Table S4). These results suggest that low expression of miR-141-3p strongly correlates with the bone metastasis of PCa.

### Upregulating miR-141-3p inhibits bone metastasis of PC-3 cells in vivo

**Fig. 2:**
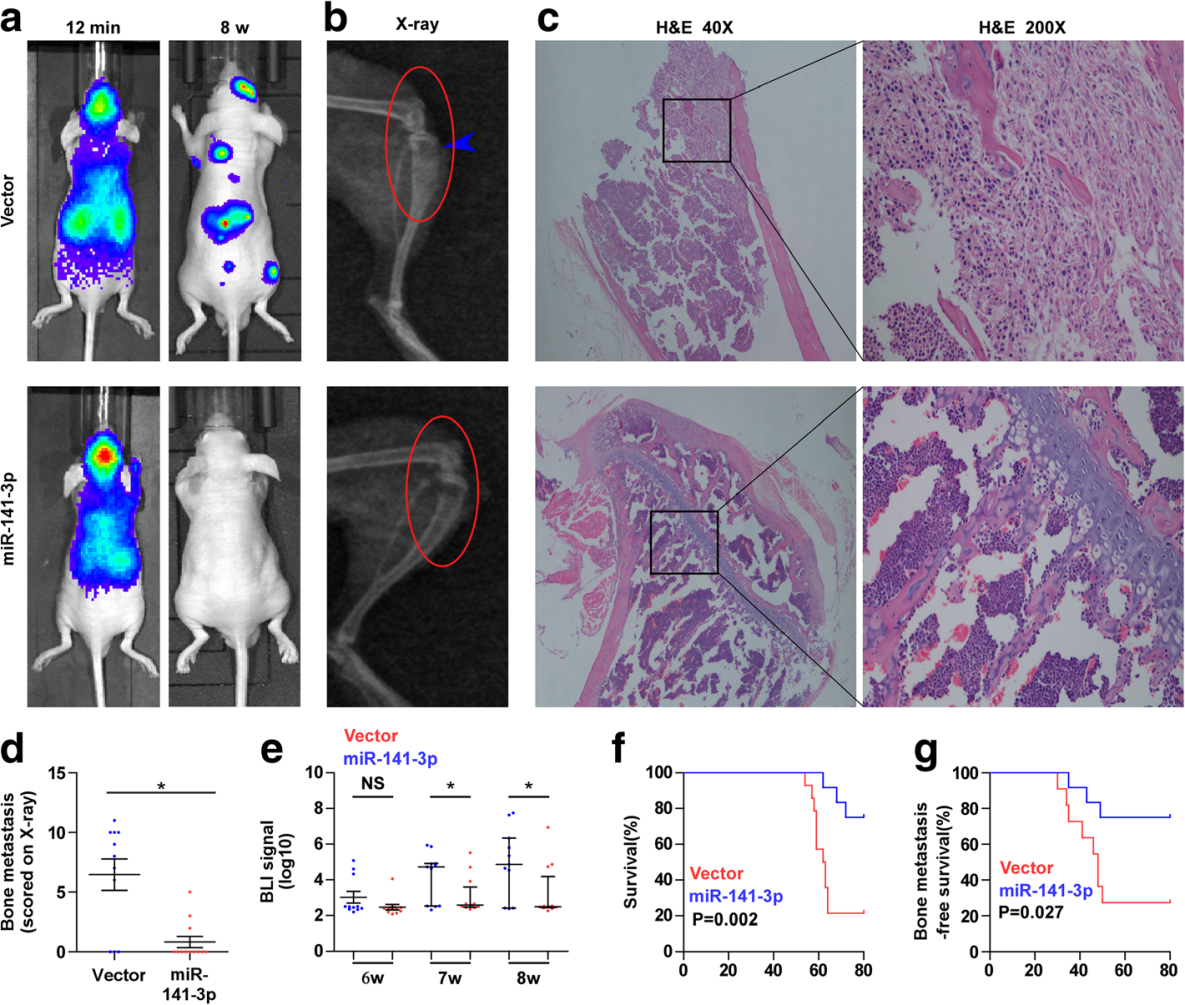
Upregulation of miR-141-3p inhibits bone metastasis of PC-3 cells in vivo. **a** Representative BLIs signal of bone metastasis of a mouse from the vector or miR-141-3p-overexpressing groups of mice at 12 mins and 8 week respectively. **b** Representative radiographic images of bone metastases in the indicated mice (arrows indicate osteolytic lesions).**c** Representative H&E-stained sections of tibias from the indicated mouse. **d** The sum of bone metastasis scores for each mouse in tumor-bearing mice inoculated with vector (n = 11) or miR-141-3p-overexpressing (*n* = 12) cells. **e** Quantification of the BLI signaling in the vector and miR-141-3p-overexpressing groups at 6, 7 and 8 weeks respectively. *P < 0.05. **f** and **g** Kaplan-Meyer analysis of mouse survival (**f**) and bone metastasis-free survival (**g**) in the vector and miR-141-3p-overexpressing groups

To determine the effect of miR-141-3p on the bone metastasis of PCa in vivo, we exogenously overexpressed miR-141-3p via virus transduction in PC-3 cells that expressed the low expression level of miR-141-3p as shown in Fig. 1e (Additional file 5: Figure S1). The luciferase-labeled vector or miR-141-3p-overexpressing PC-3 cells were inoculated respectively into the left cardiac ventricle of male nude mice to monitor the progression of bone metastasis by bioluminescence imaging (BLI). As shown in Fig. 2a and b, the miR-141-3p-overexpressing PC-3 cells presented lower bone metastasis ability compared with the control group by X-ray and BLI. H&E staining of the bone tumor sections revealed that upregulating miR-141-3p reduced the tumor burden in bone (Fig. 2c). Moreover, cells with miR-141-3p overexpression exhibited fewer bone metastatic sites and smaller osteolytic area of metastatic tumors, as well as longer survival and bone metastasis-free survival compared to the control group (Fig. 2d-g). Collectively, these finding demonstrate that upregulating miR-141-3p inhibits the bone metastasis of PCa in vivo.

### Ectopic expression of miR-141-3p inhibits EMT in PCa cells

**Fig. 3:**
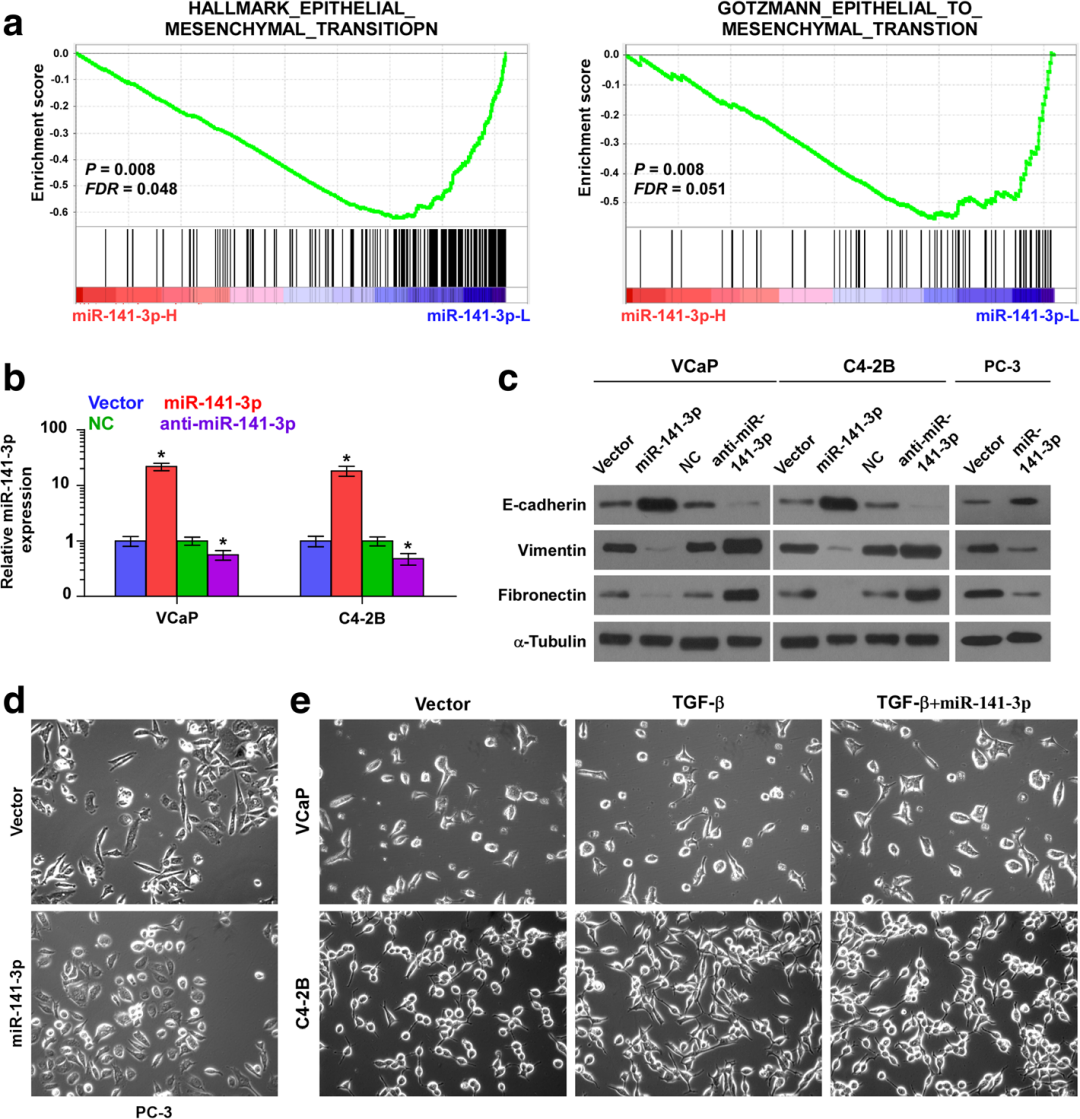
Upregulation of miR-141-3p inhibits EMT in PCa cells. **a** Gene set enrichment analysis (GSEA) revealed that low expression of miR-141-3p expression significantly and positively correlated with the EMT signatures. **b** Real-time PCR analysis of miR-141-3p expression inVCaP and C4-2B cells transduced with miR-141-3p or transfected with anti-miR-141-3p compared to controls. Transcript levels were normalized by *U6* expression. Error bars represent the mean ± s.d. of three independent experiments. *P < 0.05. **c** Overexpression of miR-141-3p increased E-cadherin expression and decreased Vimentin and Fibronectin expression in PCa cells; while silencing miR-141-3p decreased E-cadherin expression and increased Vimentin and Fibronectin expression in VCaP and C4-2B cells. α-Tubulin served as the loading control. **d** Upregulating miR-141-3p converted a stick-like or long spindleshaped mesenchymal profile to a cobblestone-like or a short spindle-shaped epithelial morphology in PC-3 cells. **e** Upregulating miR-141-3p converted a stick-like or long spindleshaped mesenchymal profile to a cobblestone-like or a short spindle-shaped epithelial morphology in VCaP and C4-2B cells treated with TGF-β (5 ng/ml for 72 h)

The specific biological role of miR-141-3p in bone metastasis of PCa was further analyzed by Gene Set Enrichment Analysis (GSEA) based on miRNA expression data from TCGA, and the result showed that low expression of miR-141-3p significantly and positively correlated with epithelial and mesenchymal transition (EMT)-associated gene signatures (Fig. 3a). We further overexpressed and downregulated miR-141-3p via transient transfection in VCaP and C4-2B cells that express moderate level of miR-141-3p (Fig. 3b). Western blot analysis showed that upregulating miR-141-3p reduced the expression of mesenchymal marker vimentin and fibronectin, and enhanced the expression of epithelial marker E-cadherin in PCa cells; conversely, silencing miR-141-3p increased vimentin and fibronectin expression, and decreased E-cadherin expression (Fig. 3c). The effect of miR-141-3p on the morphology of PCa cells was investigated and the result showed that upregulating miR-141-3p converted the stick-like or long spindle shaped mesenchymal phenotype to an evident short spindle-shaped or cobblestone-like epithelial profile in PC-3 cells (Fig. 3d). Furthermore, upregulating miR-141-3p reversed the scattered spindle-shaped morphology induced by TGF-β in VCaP and C4-2B cells (Fig. 3e). miR-141-3p expression was not affect by TGF-β treatment (Additional file 6: Figure S2). These results indicate that miR-141-3p inhibits EMT in PCa cells.

### Upregulating miR-141-3p represses migration and invasion abilities in PCa cells

**Fig. 4:**
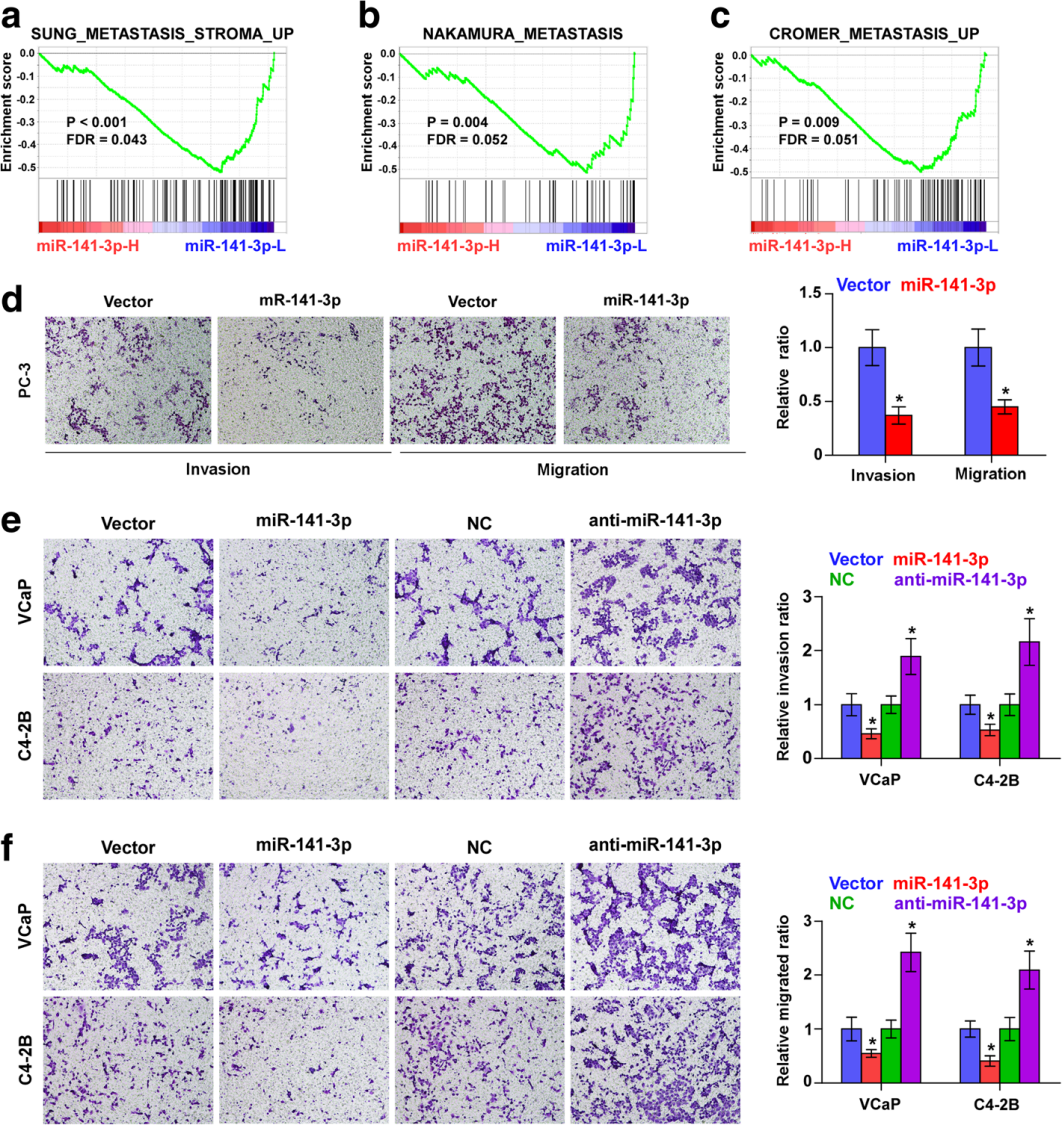
Upregulation of miR-141-3p represses invasion and migration abilities of PCa cells in vitro. **a**-**c** GSEA revealed that low expression of miR-141-3p expression significantly correlated with metastatic propensity. **d** Upreulating miR-141-3p suppressed invasion and migration abilities in PC-3 cells. Error bars represent the mean ± S.D. of three independent experiments. *P < 0.05. **e** and **f** Overexpression of miR-141-3p inhibited, while silencing miR-141-3p enhanced invasion (**e**) and migration (**f**) abilities in VCaP and C4-2B cells. Error bars represent the mean ± S.D. of three independent experiments. *P < 0.05

GSEA analysis further revealed that downexpression of miR-141-3p positively correlated with metastatic propensity (Fig. 4a-c). Then, we further evaluated the effects of miR-141-3p on invasion and migration abilities in PCa cells. As shown in Fig. 4d-f, upregulating miR-141-3p inhibited the invasion and migration abilities of PCa cells. Conversely, silencing miR-141-3p enhanced the invasion and migration abilities of PCa cells. These results indicate that miR-141-3p inhibits invasion and migration abilities in PCa cells.

### miR-141-3p targets TRAF5 and TRAF5

**Fig. 5:**
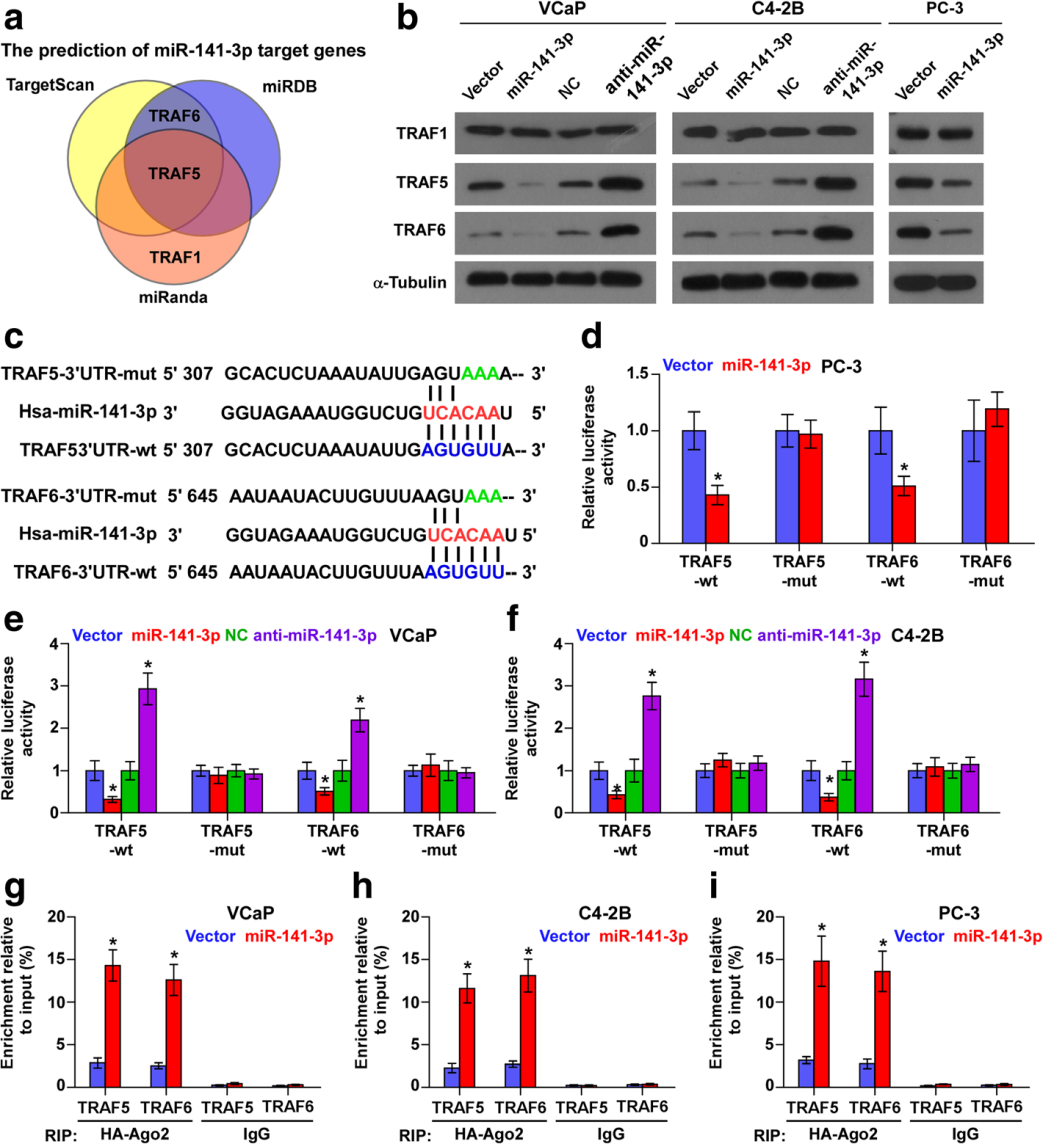
miR-141-3p targets TRAF5 and TRAF6. **a** Predictive target genes of miR-141-3p from TargetScan, miRanda and miRDB. **b** Western blotting of TRAF1, TRAF5 and TRAF6 expression in the indicated cells. α-Tubulin served as the loading control. **c** Predicted miR-141-3p targeting sequence and mutant sequences in 3’UTR s of TRAF5 and TRAF6. **d**-**f** Luciferase assay of cells transfected with pmirGLO-3’UTR reporter of TRAF5 and TRAF6 in the miR-141-3p overexpressing and silencing PCa cells. Error bars represent the mean ± S.D. of three independent experiments. *P < 0.05. **g**-**i** MiRNP IP assay showing the association between miR-141-3p and TRAF5 and TRAF61transcripts in PCa cells cells. Pulldown of IgG antibody served as the negative control. Error bars represent the mean ± S.D. of three independent experiments. *P < 0.05

Through analyzing the publicly available algorithms TargetScan and miRanda, we found that several TRAFs members, including TRAF1, TRAF5 and TRAF6, may be potential targets of miR-141-3p (Fig. 5a). RT-PCR and western blotting analysis revealed that upregulating miR-141-3p reduced, while silencing miR-141-3p increased the expression levels of TRAF5 and TRAF6, but not of TRAF1 in PCa cells (Fig. 5b and Additional file 7: Figure S3A-C). Moreover, luciferase assay revealed that upregulating miR-141-3p repressed, while silencing miR-141-3p elevated the reporter activity driven by the 3’UTRs of TRAF5 and TRAF6, but not by the mutant 3’UTR of TRAF5 and TRAF6 within the miR-141-3p–binding seed regions in PCa cells (Fig. 5c-f). miRNA immunoprecipitation (RIP) assay showed a direct association of miR-141-3p with TRAF5 and TRAF6 transcripts (Fig. 5g-i). Importantly, individual silencing of TRAF5 and TRAF6 abrogated the stimulatory effects of silencing miR-141-3p on invasion and migration abilities in PCa cells (Additional file 7: Figure S3D and E). Therefore, our results demonstrate that miR-141-3p directly targets TRAF5 and TRAF6 in PCa cells.

### miR-141-3p suppresses NF-kB activity in PCa cells

**Fig. 6:**
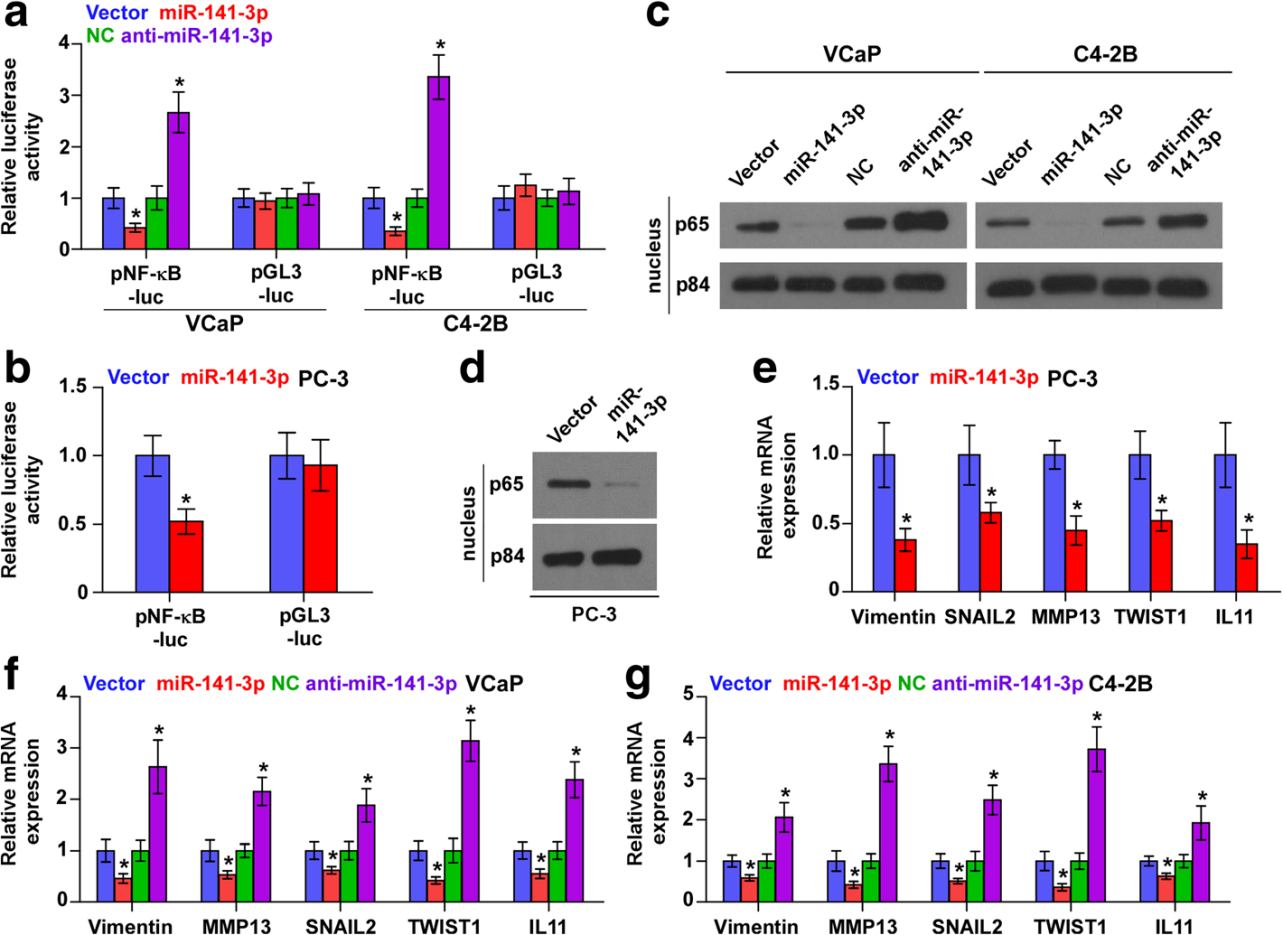
miR-141-3p inhibits NF-κB signaling pathway. **a** and **b** NF-κB transcriptional activity was assessed by luciferase reporter constructs in the indicated cells. Error bars represent the mean ± S.D. of three independent experiments. *P < 0.05. **c** and **d** Western blotting of nuclear NF-κB/p65 expression in the indicated cells. The nuclear protein p84 was used as the nuclear protein marker. **e**-**g** Real-time PCR analysis of Vimentin, TWIST1, SNAIL2, MMP13 and IL11 in the indicated cells. Transcript levels were normalized to *U6* expression. Error bars represent the mean ± S.D. of three independent experiments. *P < 0.05

As an adaptor protein of NF-kB signaling, TRAFs protein family transducer signal via binding to tumor necrosis factor (TNF) receptor cytoplasmic domains and mediating TNF-induced activation of NF-kB signaling [14, 15]. Thus, we further investigated whether miR-141-3p had an effect on the activity of NF-kB signaling. As shown in Fig. 6a and b, miR-141-3p overexpression inhibited, while silencing miR-141-3p increased NF-κB-dependent luciferase activity in PCa cells. Moreover, cellular fractionation and western blotting analysis revealed that overexpression of miR-141-3p decreased, while silencing miR-141-3p promoted nuclear accumulation of NF-κB/p65 (Fig. 6c and d). Real-time PCR analysis showed that upregulating miR-141-3p inhibited the expression levels of multiple NF-κB signaling downstream metastasis-related target genes, including Vimentin, SNAIL2, MMP13 TWIST1 and IL11 in PCa cells. Conversely, silencing miR-141-3p enhanced expression levels of these genes in PCa cells (Fig. 6e-g). Furthermore, upregulating TRAF5, TRAF6 or both partially rescued the NF-κB activity, and Vimentin, SNAI2 and TWIST1 expression repressed by miR-141-3p-overexpression in PCa cells (Additional file 8: Figure S4A-D). Thus, these results reveal that miR-141-3p inhibits NF-κB signaling pathway via targeting TRAF5 and TRAF6 in PCa cells.

### NF-κB activation is essential for the pro-metastasis role of miR-141-3p downexpression in PCa cells

We further explored whether NF-κB signaling activity mediated the pro-metastasis role of silencing miR-141-3p in PCa cells. As shown in Additional file 9: Figure S5A, the stimulatory effects of miR-141-3p silencing on NF-κB activity were attenuated by LY2409881 and JSH-23 in PCa cells. Moreover, inhibition of NF-κB signaling by LY2409881 and JSH-23 abrogated the stimulatory effects of miR-141-3p downexpression on migration and invasion abilities in PCa cells (Additional file 9: Figure S5B and C). These results indicate that NF-κB signaling activation is essential for the pro-metastasis role of miR-141-3p silencing in PCa cells.

### Clinical correlation of miR-141-3p with TRAF5, TRAF6 and NF-κB activation in human PCa tissues

**Fig. 7:**
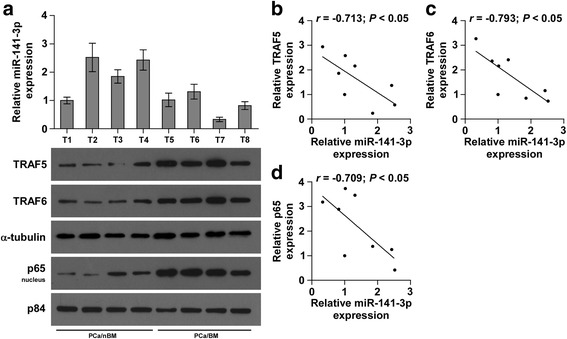
Clinical relevance of miR-141-3p with TRAF5, TRAF6 and NF-kB signaling activity in human PCa and bone tissues. **a** Analysis of miR-141-3p expression with TRAF5, TRAF6 and nuclear p65 in 4 non-bone metastatic PCa tissues (T1–4) and 4 bone metastatic PCa tissues (T5–8). U6 was used as the control for RNA loading. miR-141-3p expression levels were normalized to that miR-141-3p expression of sample one. Each bar represents the mean ± SD of three independent experiments. Loading controls were α-tubulin and p84 for the cytoplasmic and nuclear fractions. **b**-**d** Correlation between miR-141-3p levels and TRAF5, TRAF6 and nuclear p65 expression in PCa and bone tissues.The expression levels of TRAF5, TRAF6 and nuclear p65 were quantified by densitometry using Image J Software, and normalized to the levels of α-tubulin and p84, respectively. The sample 1 was used as a standard. The relative expressions of miR-141-3p and these proteins were used to perform the correlation analysis

To further investigate the clinical significance of miR-141-3p-induced TRAF5 and TRAF6 downregulation and the subsequent activation of NF-κB signaling in PCa tissues, miR-141-3p expression and the protein expression levels of TRAF5, TRAF6 and nuclear p65 were examined. As shown in Fig. 7a, miR-141-3p expression in bone metastatic PCa tissues (T5–8) was reduced compared with that in non-bone metastatic PCa tissues (T1–4) (Fig. 7a). Conversely, protein expression of TRAF5, TRAF6 and p65 expression was elevated in bone metastatic PCa tissues compared with that in non-bone metastatic PCa tissues (Fig. 7a). Pearson analysis revealed that miR-141-3p expression inversely correlated with TRAF5, TRAF6 and nuclear p65 expression (Fig. 7b-d). Consistently, miR-141-3p levels in PCa tissues were negatively associated with mRNA levels of the NF-κB signaling downstream genes MMP13, TWIST1 and IL11 (Additional file 10: Figure S6A-C).Taken together, expression level of miR-141-3p negatively correlates with TRAF5, TRAF6 and NF-κB activation in clinical PCa tissues.

## Discussion

The primary results of the current study provide novel visions into the critical role of miR-141-3p in the repressive activation of NF-κB signaling, which further inhibits bone metastasis of PCa. Here, we reported that miR-141-3p expression was reduced in bone metastatic PCa tissues, and low expression of miR-141-3p correlated with PSA levels, Gleason grade and bone metastasis status in PCa patients. Our results further indicate that miR-141-3p inhibits NF-κB signaling in PCa cells via directly targeting TRAF5 and TRAF6, which further suppresses bone metastasis of PCa. Therefore, our results indicate miR-141-3p plays a tumor suppressive role in bone metastasis of PCa via inhibiting NF-κB signaling.

Extensive studies have shown that NF-κB signaling was constitutively activated in a variety of human cancer types and is associated with tumor initiation, progression and metastasis [6, 37]. Helbig and colleagues has demonstrated that NF-kappaB promoted the motility of breast cancer cells by transcriptionally up-regulating the expression of CXCR4 [38]; furthermore, aberrant activation of NF-κB signaling regulates multiple genes expression, including VEGF and IL-8, which are important for lung tumorigenesis via induction of angiogenesis [39]. Accumulating literatures have demonstrated that activation NF-κB signaling is essential for the bone metastasis of cancers [16, 19]. Park et al. reported that constitutive activation of NF-κB signaling in breast cancer cells promoted the bone resorption characteristic of osteolytic bone metastasis. The mediating gene involved in osteolytic bone metastasis of breast cancer was a key target of NF-κB signaling: granulocyte macrophage-colony stimulating factor (GM-CSF) promoted osteolytic bone metastasis of breast cancer cells by stimulating osteoclast development [17]. Importantly, Chen et al. reported that NF-κB signaling was crucial for the development of PCa bone metastasis [19]. However, the underlying mechanism of constitutive activation of NF-κB signaling in the bone metastasis of PCa remains poorly known. In this study, our results revealed that TRAF5 and TRAF6 were direct targets of miR-141-3p in PCa cells. In turn, downexpression of miR-141-3p constitutively activated NF-κB signaling through upregulating TRAF5 and TRAF6 in PCa cells. Importantly, inhibition of NF-κB signaling activity by specific inhibitors of NF-κB signaling LY2409881 and JSH-23attenuated the stimulatory effects of silencing miR-141-3p on invasion and migration of PCa cells. Taken together, our results uncover a novel regulatory mechanism for NF-kB activation in bone metastasis of PCa.

As an adaptor protein of NF-kB signaling cascades, TRAFs protein family transduct signal via binding to tumor necrosis factor (TNF) receptor cytoplasmic domains and mediating TNF-induced activation of NF-kB signaling [14, 15]. Accumulating literatures have demonstrated that overexpression of TRAFs promotes the progression and aggressiveness of cancers via activating NF-kB signaling. For example, TRAF2 has been reported to upregulated in 15% of human epithelial cancer due to amplification and rearrangement, which contributes to the constitutive activation of NF-kB signaling [40]. Compagno et al. reported that somatic mutation of TRAF5 sustained the activity of NF-kB signaling, which was associated with most aggressive subtype, activated B-cell-like Diffuse large B-cell lymphoma [41]. Moreover, a study by Starczynowski and colleagues showed that TRAF6 exhibited concomitant mRNA overexpression and gene amplification in RAS-driven lung cancers. TRAF6 overexpression in NIH3T3 cells promoted anchorage-independent growth and tumor formation via activating NF-κB signaling [42]. Thus, further exploring the mechanisms of regulation of TRAFs would increase our knowledge of the biologic basis of the constitutive activation of NF-kB in cancer and provide novel insights for tumor therapy. In this study, our results found that miR-141-3p directly suppressed the expression of TRAF5 and TRAF6, which further constrained the NF-kB signaling activity. Therefore, our results indicate that miR-141-3p inactivates NF-kB signaling via targeting TRAF5 and TRAF6, which further inhibits bone metastasis of PCa.

Abundant studies have shown that miR-141-3p was downexpressed in multiple human cancers, including hepatocellular carcinoma, prostate cancer, breast cancer, renal cell carcinoma, pancreatic cancer and gastric cancer and that low expression of miR-141-3p promoted cancer cell invasion and metastasis via varying mechanisms [29, 30, 43–46]. However, recent literatures reported that miR-141-3p was upregulated in nasopharyngeal carcinoma and acted as an oncogenic miRNA [47, 48]. These studies indicate that the pro- and anti-cancer roles of miR-141-3p are tumor-type dependent. Furthermore, low expression of miR-141-3p has been reported to be closely associated with metastatic phenotype of cancers [49, 50]. Importantly, miR-141-3p expression levels has been reported to be downexpressed in bone metastatic PCa cells PC-3 [29], suggesting that low expression of miR-141-3p plays an important role in the bone metastasis of PCa. However, the clinical significance and biological roles of miR-141-3p in bone metastasis of PCa remain largely unknown. In this study, our results revealed that miR-141-3p expression was reduced in human bone metastatic PCa tissues and cells. Low expression of miR-141-3p was positively associated with serum PSA level, Gleason grade and distant bone metastasis status in PCa patients. Our results further revealed that miR-141-3p repressed the activity of NF-κB signaling via targeting TRAF5 and TRAF6, which further inhibited the EMT, invasion, migration and bone metastasis of PCa cells in vitro and in vivo. Collectively, our findings indicate that miR-141-3p plays an important role in the bone metastasis of PCa.

## Conclusions

In summary, our results demonstrate that downexpression of miR-141-3p promotes the development of bone metastasis in PCa via upregulating TRAF5 and TRAF6, resulting in the sustained activity of NF-κB signaling. Thus, in-depth of understanding the specific role of miR-141-3p in the pathogenesis of PCa bone metastasis will facilitate the development of novel therapeutic methods for the treatment of PCa bone metastasis.

## Additional files

Additional file 1: Table S1. A list of primers used in the reactions for clone PCR. (PDF 6 kb)

Additional file 2: Table S2. A list of primers used in the reactions for real-time RT-PCR. (PDF 10 kb)

Additional file 3: Table S3. The clinicopathological characteristics in 141 patients with prostate cancer. (PDF 52 kb)

Additional file 4: Table S4. The relationship between miR-141-3p and clinicopathological characteristics in 141 patients with prostate cancer. (PDF 57 kb)

Additional file 5: Figure S1. Real-time PCR analysis of miR-141-3p expression in PC-3 cells transduced with pre-miR-141 compared to controls. Transcript levels were normalized by *U6* expression. Error bars represent the mean ± s.d. of three independent experiments. *P < 0.05. (PDF 20 kb)

Additional file 6: Figure S2. Real-time PCR analysis of miR-141-3p expression in VCaP and C4-2B cells treated with TGF-β (5 ng/ml for 48 h). Transcript levels were normalized by *U6* expression. Error bars represent the mean ± s.d. of three independent experiments. *P < 0.05. (PDF 30 kb)

Additional file 7: Figure S3. (**A**-**C**) Real-time PCR analysis of TRAF1, TRAF5 and TRAF6 expression in the indicated cells. Error bars represent the mean ± S.D. of three independent experiments. *P < 0.05. (**D** and **E**) Individual silencing of TRAF5 and TRAF6 attenuated the stimulatoy effects of silencing miR-141-3p on the invasion (**D**) and migration (**E**) abilities in PCa cells. *P < 0.05. (PDF 123 kb)

Additional file 8: Figure S4. The effects of miR-141-3p on NF-kB activity and Vimentin, SNAI2 and TWIST1 expression are TRAF targeting dependent in PCa cells (**A**) Upregulating TRAF5, TRAF6 or both partially rescued the NF-kB activity repressed by miR-141-3p-overexpression in PCa cells. *P < 0.05 and **P < 0.01. (**B**-**D**) Real-time PCR analysis revealed that upregulating TRAF5, TRAF6 or both partially rescued the Vimentin, SNAI2 and TWIST1 expression repressed by miR-141-3p-overexpression in PCa cells. *P < 0.05 and **P < 0.01. (PDF 115 kb)

Additional file 9: Figure S5. NF-κB activation is essential for the pro-metastasis role of miR-141-3p downexpression in PCa cells (**A**) NF-κB signaling inhibitors LY2409881 (10 μM) and JSH-23 (10 μM) attenuated the stimulatory effect of silencing miR-141-3p on NF-κB transcriptional activity in the indicated cells respectively. Error bars represent the mean ± s.d. of three independent experiments. *P < 0.05. (**B**) NF-κB signaling inhibitors LY2409881 (10 μM) and JSH-23 (10 μM) attenuated the stimulatory effect of silencing miR-141-3p on invasion ability in the indicated cells respectively. Error bars represent the mean ± s.d. of three independent experiments. *P < 0.05. (**C**) NF-κB signaling inhibitors LY2409881 (10 μM) and JSH-23 (10 μM) attenuated the stimulatory effect of silencing miR-141-3p on migration ability in the indicated cells respectively. Error bars represent the mean ± s.d. of three independent experiments. *P < 0.05 and **P < 0.01. (PDF 82 kb)

Additional file 10: Figure S6. Clinical relevance of miR-141-3p with the expression of downstream genes of NF-κB signaling in PCa tissues. (**A**-**C**) Correlation analysis of miR-141-3p expression and MMP13, TWIST1 and IL11 mRNA expression levels in 8 clinical PCa tissues. (PDF 78 kb)

## Abbreviations

EMT: Epithelial-mesenchymal transition
H&E: Hematoxylin and Eosin Stain
IL11: Interleukin 11
miR-141-3p: miRNA-141-3p
MMP13: Matrix Metallopeptidase 13
NF-κB: nuclear factor kappa-light-chain-enhancer of activated B cells
PCR: Polymerase Chain Reaction
SNAIL2: Snail family transcriptional repressor 2
TCGA: The Cancer Genome Atlas
TRAF1: Tumor necrosis factor receptor-associated factor 1
TRAF5: Tumor necrosis factor receptor-associated factor 5
TRAF6: Tumor necrosis factor receptor-associated factor 6
TWIST1: Twist family bHLH transcription factor 1
