# Supplementary material for: Downregulation of miR-141-3p promotes bone metastasis via activating NF-κB signaling in prostate cancer
Source: J Exp Clin Cancer Res. 2017 Dec 4;36:173. doi: 10.1186/s13046-017-0645-7 (PMC5716366; doi:10.1186/s13046-017-0645-7)
Supplement: Supplementary file 1 — A list of primers used in the reactions for clone PCR. (PDF 6 kb) [file 13046_2017_645_MOESM1_ESM.pdf]

Table S1. A list of primers used in the reactions for clone PCR.

**Used for subcloning and plasmid construction:**	
miR-141-3p-clone-Forward	TCCCACCCAGTGCGATTTGTC
miR-141-3p-clone-Reverse	GTTGCTGGGAGGCTAAGATGAG
TRAF5-3UTR-clone-Forward	ACTGTGGAGGAGAGCACATTTG
TRAF5-3UTR-clone-Reverse	CTTGCAAAACTGTGCTTGCAAAC
TRAF6-3UTR-clone-Forward	AGTGCCTTTCCTTGCCCTGTTC
TRAF6-3UTR-clone-Reverse	GGGGAAGATGCTACTTCGTAACCTC
