# Supplementary material for: Downregulation of miR-141-3p promotes bone metastasis via activating NF-κB signaling in prostate cancer
Source: J Exp Clin Cancer Res. 2017 Dec 4;36:173. doi: 10.1186/s13046-017-0645-7 (PMC5716366; doi:10.1186/s13046-017-0645-7)
Supplement: Supplementary file 2 — A list of primers used in the reactions for real-time RT-PCR. (PDF 10 kb) [file 13046_2017_645_MOESM2_ESM.pdf]

Table S2. A list of primers used in the reactions for real-time RT-PCR.

**Real-time PCR primer:**	
TRAF1-forward	CTTCCCTTGAAGGAGCAGC
TRAF1-reverse	CTATAAGCCCAGGAAGCCG
TRAF5-forward	GGATGAAACCACAGGGCATA
TRAF5-reverse	GCAGCCAGGAGCAGCAG
TRAF6-forward	GCCACACAGCAGTCACTTTC
TRAF6-reverse	TCCCCGCGCACTAGAAC
IL11-forward	TGAAGACTCGGCTGTGACC
IL11- reverse	CCTCACGGAAGGACTGTCTC
TWIST1-forward	TCCATTTTCTCCTTCTCTGGAA
TWIST1-reverse	GTCCGCGTCCCACTAGC
MMP13-forward	AACATCCAAAAACGCCAGAC
MMP13-reverse	GGAAGTTCTGGCCAAAATGA
Vimentin-forward	ATTCCACTTTGCGTTCAAGG
Vimentin-reverse	CTTCAGAGAGAGGAAGCCGA
SNAIL2-forward	TGACCTGTCTGCAAATGCTC
SNAIL2-reverse	CAGACCCTGGTTGCTTCAA
GAPDH-forward	ATTCCACCCATGGCAAATTC
GAPDH-reverse	TGGGATTTCCATTGATGACAAG
