# Supplementary material for: Downregulation of miR-141-3p promotes bone metastasis via activating NF-κB signaling in prostate cancer
Source: J Exp Clin Cancer Res. 2017 Dec 4;36:173. doi: 10.1186/s13046-017-0645-7 (PMC5716366; doi:10.1186/s13046-017-0645-7)
Supplement: Supplementary file 3 — The clinicopathological characteristics in 141 patients with prostate cancer. (PDF 52 kb) [file 13046_2017_645_MOESM3_ESM.pdf]

Table S3. The clinicopathological characteristics in 141 patients with prostate cancer

Parameters	Number of cases
Age (years)	
≤75	76
>75	65
Differentiation	
Well/moderate	62
Poor	79
Serum PSA at diagnosis, μg/mL	
<18.9	71
>18.9	70
Gleason grade	
≤7	77
>7	64
Operation	
TURP	57
Needle biopsy	65
TURP+PP	3
TURP+BO	10
BO	6
miR-141-3p expression	
<4.13	71
>4.13	70
BM-status	
BM-free	89
BM	52

Abbreviation: PSA, prostate-specific antigen; TURP, Trans Urethral Resection Prostate; PP, Prior Prostatectomy; BO, Bilateral Orchiectomies; SD, Standard deviation; IHC, Immunological Histological Chemistry; BM, Bone Metastasis.
