# Supplementary material for: Downregulation of miR-141-3p promotes bone metastasis via activating NF-κB signaling in prostate cancer
Source: J Exp Clin Cancer Res. 2017 Dec 4;36:173. doi: 10.1186/s13046-017-0645-7 (PMC5716366; doi:10.1186/s13046-017-0645-7)
Supplement: Supplementary file 4 — The relationship between miR-141-3p and clinicopathological characteristics in 141 patients with prostate cancer. (PDF 57 kb) [file 13046_2017_645_MOESM4_ESM.pdf]

Table S4. The relationship between miR-141-3p and clinicopathological characteristics in 141 patients with prostate cancer.

Parameters	Number of cases	miR-141-3p expression		*P* values
		Low	High	
Age (years)				
≤75	76	33	43	0.075
>75	65	38	27	
Differentiation				
Well/moderate	62	36	26	0.105
Poor	79	35	44	
Serum PSA				
<18.9	71	25	46	
>18.9	70	46	24	<0.001*
Gleason grade				
≤7	77	23	54	
>7	64	48	16	<0.001*
Operation				
TURP	57	30	27	
Needle biopsy	65	34	31	0.109
TURP+PP	3	3	0	
TURP+BO	10	2	8	
BO	6	2	4	
BM-status				
nBM	89	35	54	
BM	52	36	16	<0.001*
