# Supplementary figures and images for: Downregulation of miR-141-3p promotes bone metastasis via activating NF-κB signaling in prostate cancer
Source: J Exp Clin Cancer Res. 2017 Dec 4;36:173. doi: 10.1186/s13046-017-0645-7 (PMC5716366; doi:10.1186/s13046-017-0645-7)

Supplemental Figure 1

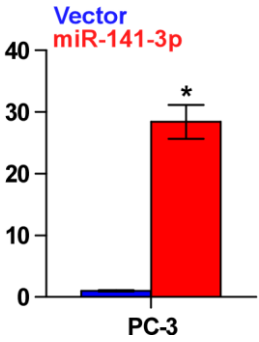

Supplement: Supplementary file 5 — Real-time PCR analysis of miR-141-3p expression in PC-3 cells transduced with pre-miR-141 compared to controls. Transcript levels were normalized by U6 expression. Error bars represent the mean ± s.d. of three independent experiments. *P < 0.05. (PDF 20 kb) [file 13046_2017_645_MOESM5_ESM.pdf]

Supplemental Figure 2

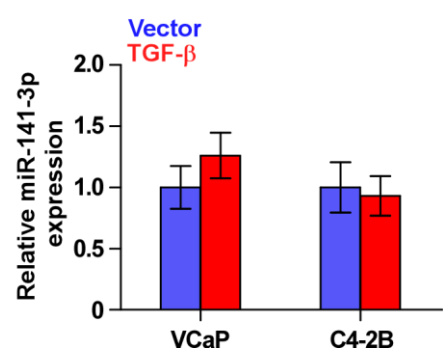

Supplement: Supplementary file 6 — Real-time PCR analysis of miR-141-3p expression in VCaP and C4-2B cells treated with TGF-β (5 ng/ml for 48 h). Transcript levels were normalized by U6 expression. Error bars represent the mean ± s.d. of three independent experiments. *P < 0.05. (PDF 30 kb) [file 13046_2017_645_MOESM6_ESM.pdf]

**Supplemental Figure 3**

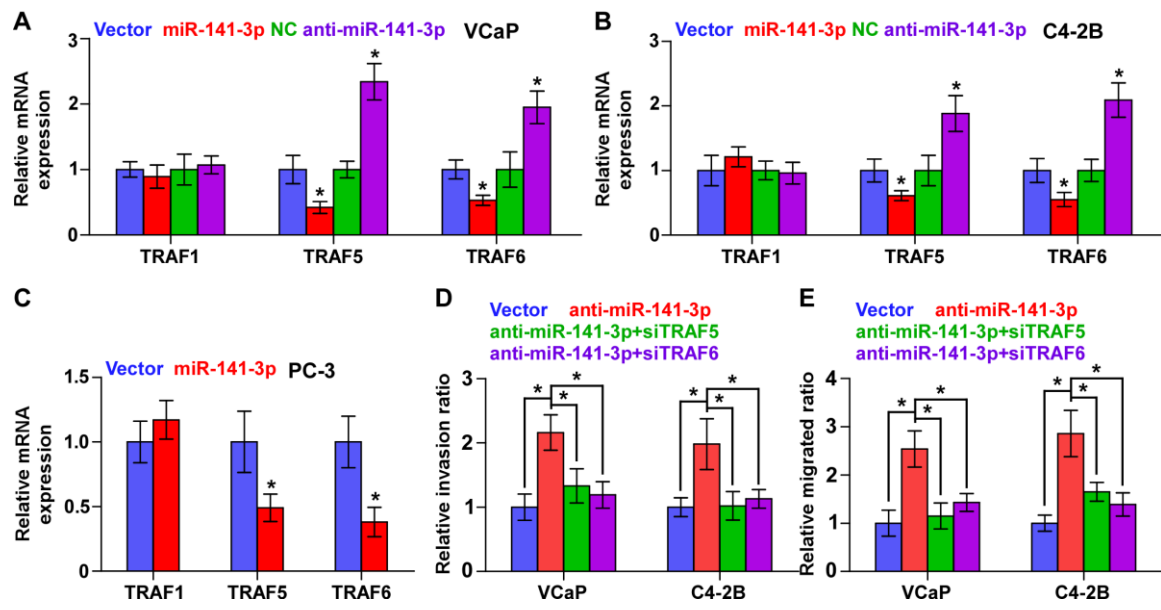

Supplement: Supplementary file 7 — (A-C) Real-time PCR analysis of TRAF1, TRAF5 and TRAF6 expression in the indicated cells. Error bars represent the mean ± S.D. of three independent experiments. *P < 0.05. (D and E) Individual silencing of TRAF5 and TRAF6 attenuated the stimulatoy effects of silencing miR-141-3p on the invasion (D) and migration (E) abilities in PCa cells. *P < 0.05. (PDF 123 kb) [file 13046_2017_645_MOESM7_ESM.pdf]

**Supplemental Figure 4**

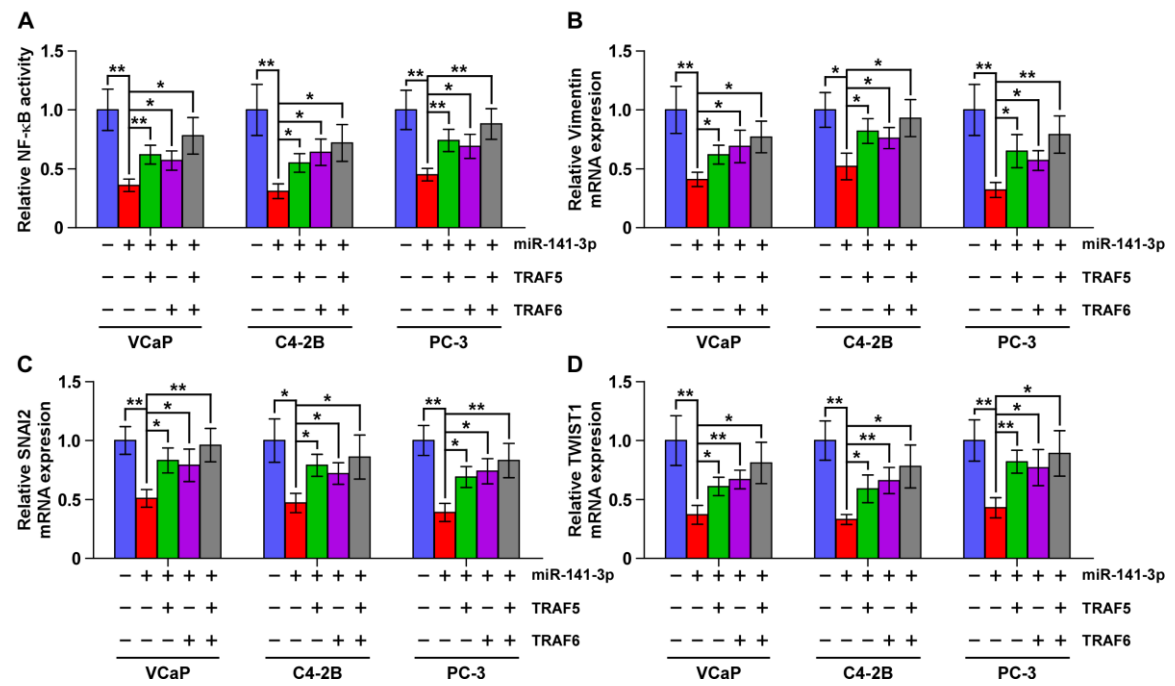

Supplement: Supplementary file 8 — The effects of miR-141-3p on NF-kB activity and Vimentin, SNAI2 and TWIST1 expression are TRAF targeting dependent in PCa cells (A) Upregulating TRAF5, TRAF6 or both partially rescued the NF-kB activity repressed by miR-141-3p-overexpression in PCa cells. *P < 0.05 and **P < 0.01. (B-D) Real-time PCR analysis revealed that upregulating TRAF5, TRAF6 or both partially rescued the Vimentin, SNAI2 and TWIST1 expression repressed by miR-141-3p-overexpression in PCa cells. *P < 0.05 and **P < 0.01. (PDF 115 kb) [file 13046_2017_645_MOESM8_ESM.pdf]

## Supplemental Figure 5

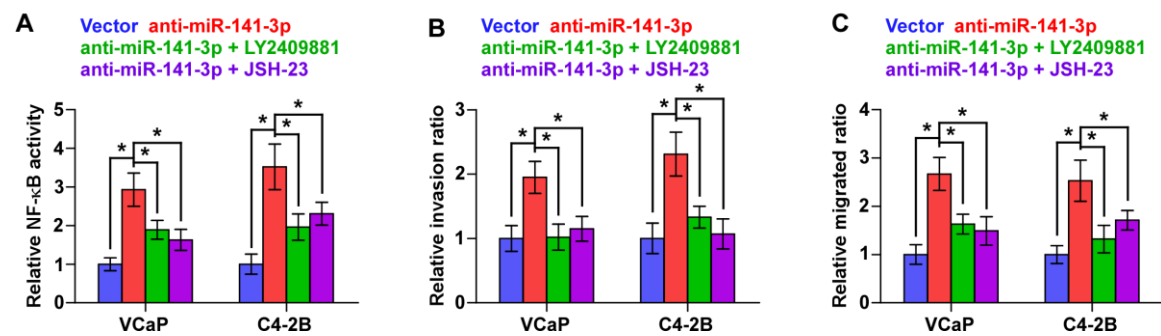

Supplement: Supplementary file 9 — NF-κB activation is essential for the pro-metastasis role of miR-141-3p downexpression in PCa cells (A) NF-κB signaling inhibitors LY2409881 (10 μM) and JSH-23 (10 μM) attenuated the stimulatory effect of silencing miR-141-3p on NF-κB transcriptional activity in the indicated cells respectively. Error bars represent the mean ± s.d. of three independent experiments. *P < 0.05. (B) NF-κB signaling inhibitors LY2409881 (10 μM) and JSH-23 (10 μM) attenuated the stimulatory effect of silencing miR-141-3p on invasion ability in the indicated cells respectively. Error bars represent the mean ± s.d. of three independent experiments. *P < 0.05. (C) NF-κB signaling inhibitors LY2409881 (10 μM) and JSH-23 (10 μM) attenuated the stimulatory effect of silencing miR-141-3p on migration ability in the indicated cells respectively. Error bars represent the mean ± s.d. of three independent experiments. *P < 0.05 and **P < 0.01. (PDF 82 kb) [file 13046_2017_645_MOESM9_ESM.pdf]

**Supplemental Figure 6**

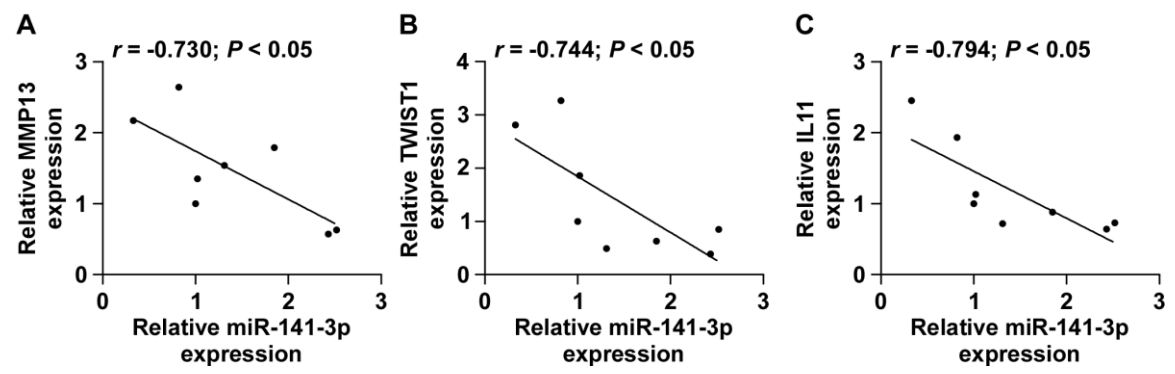

Supplement: Supplementary file 10 — Clinical relevance of miR-141-3p with the expression of downstream genes of NF-κB signaling in PCa tissues. (A-C) Correlation analysis of miR-141-3p expression and MMP13, TWIST1 and IL11 mRNA expression levels in 8 clinical PCa tissues. (PDF 78 kb) [file 13046_2017_645_MOESM10_ESM.pdf]
